# Prevalence of viral DNA in high-grade serous epithelial ovarian cancer and correlation with clinical outcomes

**DOI:** 10.1371/journal.pone.0294448

**Published:** 2023-12-01

**Authors:** Sharon E. Robertson, Maya Yasukawa, Douglas C. Marchion, Yin Xiong, Syeda Mahrukh Hussnain Naqvi, Tarik Gheit, Massimo Tommasino, Robert M. Wenham, Anna R. Giuliano, Johnathan M. Lancaster, Mian M. K. Shahzad

**Affiliations:** 1 Department of Gynecology Oncology, H. Lee Moffitt Cancer Center and Research Institute, Tampa, Florida, United States of America; 2 Department of Pathology, H. Lee Moffitt Cancer Center and Research Institute, Tampa, Florida, United States of America; 3 Department of Biostatistics and Bioinformatics, H. Lee Moffitt Cancer Center and Research Institute, Tampa, Florida, United States of America; 4 International Agency for Research on Cancer, World Health Organization, Lyon, France; 5 Department of Oncologic Sciences, University of South Florida, Tampa, Florida, United States of America; 6 Risk Assessment, Detection and Intervention Program, H. Lee Moffitt Cancer Center and Research Institute, Tampa, Florida, United States of America; Rational Vaccines Inc, UNITED STATES

## Abstract

**Introduction:**

Currently 11 infectious agents are classified as carcinogenic but the role of infectious agents on outcomes of epithelial ovarian cancer is largely unknown.

**Objective:**

To explore the association between infectious agents and ovarian cancer, we investigated the prevalence of viral DNA in primary ovarian cancer tumors and its association with clinical outcomes.

**Methods:**

Archived tumors from 98 patients diagnosed with high-grade serous epithelial ovarian cancer were collected between 1/1/1994 and 12/31/2010. After DNA extraction, Luminex technology was utilized to identify polymerase chain reaction-amplified viral DNA for 113 specific viruses. Demographic data and disease characteristics were summarized using descriptive statistics. We used logistic regression and Cox proportional hazards model to assess associations between tumor viral status and disease outcome and between tumor viral presence and overall survival (OS), respectively.

**Results:**

Forty-six cases (45.9%) contained at least one virus. Six highly prevalent viruses were associated with clinical outcomes and considered viruses of interest (VOI; Epstein-Barr virus 1, Merkel cell polyomavirus, human herpes virus 6b, and human papillomaviruses 4, 16, and 23). Factors independently associated with OS were presence of VOI (HR 4.11, *P* = 0.0001) and platinum sensitivity (HR 0.21, *P*<0.0001). Median OS was significantly decreased when tumors showed VOI versus not having these viruses (22 vs 44 months, *P*<0.0001). Women <70 year old with VOI in tumors had significantly lower median OS versus age-matched women without VOI (20 vs 57 months, *P* = 0.0006); however, among women ≥70 years old, there was no difference in OS by tumor virus status.

**Conclusions:**

The presence of a VOI was significantly associated with a lower OS. These findings may have implications for clinical management of ovarian cancer but require additional studies.

## Introduction

An association between infectious agents and cancer was first described during the early 20th century. Today, 11 infectious agents are classified as carcinogenic by the International Agency for Research on Cancer [1]. As many as 16% of the world’s new cancer cases are thought to be attributable to an infectious agent [2]. These include human papillomavirus (HPV), hepatitis B virus (HBV), hepatitis C virus, Epstein-Barr virus (EBV), human T-cell lymphotrophic virus, human herpes virus (HHV), and Merkel cell virus (MCPyV) [2]. Ovarian cancer, the most lethal gynecologic malignancy [3], does not have a known infectious etiology.

The prevalence of HPV in malignant ovarian tumors has been reported to range from 0%-66% [4–6]. Regional differences in the prevalence of HPV in malignant ovarian tumors exist, with studies from North America reporting a lower overall prevalence compared with other regions (0%-9%) [4–6]. Bilyk et al. compared the prevalence of HPV in ovarian tissue from patients at high risk of developing ovarian cancer versus normal controls and found an increased prevalence of virus among the high-risk population (40% vs 10%) [7]. However, a clear etiologic role for HPV in the development of ovarian cancer has not been demonstrated.

Evidently, HPV DNA is consistently detected in ovarian tumors; however, the presence of virus may be a post-tumor event. Kines et al. demonstrated that HPV is preferentially taken up by ovarian cancer cells compared with normal cells in culture [8]. Pandya et al. reported that the expression of viral microRNAs in ovarian cancer tissues is higher than expression in control tissues and that expression of specific microRNAs correlates with clinical outcome [9]. These data suggest that malignant ovarian tumors contain viruses, and these viruses have the potential to affect clinical outcome. Among ovarian cancer, high grade serous ovarian cancer is the most common histology. Here, we investigated the prevalence of viral DNA in high grade serous ovarian cancer in a cohort from a single institution and assessed the association between presence of viral DNA and clinical outcomes.

## Methods

### Patients’ identification and tissue extraction

This study was conducted in strict accordance with the principles expressed in the Declaration of Helsinki. The research protocol was originally reviewed and approved via expedited review by Liberty Institutional Review Board (IRB) on July 9, 2014. Liberty IRB grants waiver of informed consent. The research poses a minimal risk of harm to the subjects, it will not adversely affect the rights and welfare of the subjects, and it is not practicable to conduct the research without the waiver of informed consent. ADVARRA IRB approved a continuing review approval December, 19, 2022 via expedited review.

This study utilized the Moffitt Cancer Center Total Cancer Care (TCC®) institutional clinico-genomic tissue and data repository. All patients incorporated in the TCC protocol had provided written informed consent prior to tissue storage for future research endeavors. In this study, we obtained DNA extraction from frozen tissue samples from patients with high-grade serous epithelial ovarian cancer (SEOC). These samples were originally collected during primary cytoreductive surgery between January 1, 1994, and December 31, 2010. Tissue samples from tumors that had been exposed to any form of chemotherapeutic agents before surgery, non-serous, low-grade or borderline histology were excluded from the study. In May 2015, DNA was extracted from the frozen Formalin-fixed paraffin-embedded (FFPE) tissues. The microtome was cleaned prior to use. The microtome blade was changed between samples. The tech wore gloves and wiped them down between samples. Three at 10 um sections were used for each FFPE block for DNA extraction. Scrolls were cut directly into sterile nucleic acid free vials. DNA was extracted using the Qiagen DNeasy kit by manufacturer guidelines. DNA was subsequently shipped directly to the International Agency for Research on Cancer (IARC) for further analysis. Chart abstraction was also performed to collect demographic data (age at cancer diagnosis and race/ethnicity), disease characteristics (cancer stage, presence of ascites, pleural effusion, and lymph node metastasis), treatment characteristics (status of surgical cytoreduction, response to first-line chemotherapy, and platinum-sensitive disease), and survival data.

### Virus identification

As previously mentioned, virus identification was performed at IARC. FFPE SEOC tissue blocks were obtained from Moffitt’s tissue repository. Genomic DNA (400 ng) was extracted from these specimens using standard techniques. DNA was amplified by a multiplex polymerase chain reaction (PCR) protocol and identified as belonging to one of 113 infectious agents, including 93 HPVs, 10 polyomaviruses, and 8 herpesviruses, as well as the bacterium *Chlamydia trachomatis* using Luminex technology [10–14].

The multiplex type-specific PCR method uses specific primers for the detection of 19 probable/possible high-risk or high-risk alpha HPV types (HPV16, 18, 26, 31, 33, 35, 39, 45, 51, 52, 53, 56, 58, 59, 66, 68a and b, 70, 73, and 82); 2 low-risk alpha HPV types (HPV6 and 11); 43 beta HPV types (HPV5, 8, 9, 12, 14, 15, 17, 19, 20, 21, 22, 23, 24, 25, 36, 37, 38, 47, 49, 75, 76, 80, 92, 93, 96, 98, 99, 100, 104, 105, 107, 110, 111, 113, 115, 118, 120, 122, 124, 143, 145, 150, and 151); 29 gamma HPV types (HPV4, 48, 50, 60, 65, 88, 95, 101, 103, 108, 109, 112, 116, 119, 121, 123, 126, 127, 128, 129, 130, 131, 132, 133, 144, 148, 149, 156, and SD2) [15]; 12 polyomaviruses (BKPyV, KIPyV, WUPyV, JCPyV, HPyV6, HPyV7, HPyV9, MCPyV, TSPyV, HPyV9, HPyV10, HPyV12, and SV40); and 8 herpesviruses (HSV1, HSV2, HHV3, EBV1 and 2, cytomegalovirus, HHV6a and b, HHV7, and HHV8). Two primers for the amplification of beta -globin were also added to provide a positive control for the quality of the template DNA.

After PCR amplification, 10 μL of each reaction were analyzed by multiplex genotyping using a Luminex-based assay as previously described in detail [14, 16].

### Statistical analyses

Statistical analyses were performed using SAS 9.4 and R version 3.4.0 software. Descriptive statistics were performed for demographic data and disease characteristics. Logistic regression was performed to assess the association between the presence of viral DNA and clinical characteristics. Cox proportional hazard model was used to assess the independent association between viral DNA presence and overall survival (OS). A backward model selection was used for both logistic regression and the Cox proportional hazard models. All variables significant at *P* ≤ 0.1 remained in the final model.

## Results

### Prevalence of virus

We initially identified 101 cases of high-grade epithelial ovarian cancer. After exclusion of 3 samples that were non-serous histology, 98 samples were available for analysis, with 46 of these specimens (46.9%) containing DNA from at least one virus. Multiple viral infections were found in one tumor specimen that tested positive for two beta HPV types (HPV23 and HPV111), one gamma HPV type (HPV123), and one herpesvirus (HHV6B). Two herpesviruses (EBV1 and HHV6b), one polyomavirus (MCPyV), one gamma HPV type (HPV4), one beta HPV type (HPV23), and one mucosal high-risk HPV type 16 (HPV16) were the six most prevalent viruses.

DNA from 5 of the prevalent viruses (EBV1, HHV6B, MCPyV, HPV4, and HPV16) were each identified in four unique tumor specimens (4.1%), whereas HPV23 viral DNA was identified in nine tumor specimens (9.2%). Preliminary survival analyses suggested that patients with tumor samples containing one or more of the highly prevalent viruses had significantly worse OS than patients with tumors containing viral DNA that was not highly prevalent or tumors without any viral DNA. These highly prevalent viruses (EBV1, MCPyV, HHV6b, HPV4, HPV16, and HPV23) were therefore grouped and considered viruses of interest (VOI) for the purposes of subsequent analyses. The prevalence of VOI in the SEOC specimens was 24.5%.

### Clinical characteristics

Table 1 presents the clinical characteristics of the patient population and the association of VOI. Most of the SEOC specimens (95%) were obtained from patients with white ethnicity, with 88% of patients having stage III or IV disease at diagnosis. Most patients had an initial optimal cytoreductive surgery (72%) and platinum-sensitive disease (64%). Optimal cytoreduction was defined as no residual tumor or tumor < 1 cm at the completion of surgery, and platinum-sensitive disease was defined as recurrent disease 6 months or more from the completion of prior platinum-based chemotherapy. Older age (≥ 70 years) was the only clinical variable significantly associated with the presence of a VOI in univariate analyses. Multivariate logistic regression analysis confirmed a significant association (odds ratio 4.70; 95% confidence interval, 1.48–14.97) between age ≥ 70 years and presence of VOI in a specimen (Table 1).

**Table 1:** Demographic and disease characteristics for study population.

Variable	Numbers Total N = 98 N, (%)	Odds Ratio (95%CI)	P Value
**Race** White Non White	92, (93.9) 6, (6.1)	0.27 (0.03- 2.10)	0.2122
**Age** < 70 years old > = 70 years old	69, (70.4) 29, (29.6)	4.70 (1.48–14.97)	**0.009**
**Stage** I&II III & IV	12, (12.2) 86, (87.8)	1.88 (0.21–16.82)	0.571
**Ascites** Absent Present	64, (65.3) 33, (33.7)	0.99 (0.31–3.08)	0.987
**Pleural Effusion** Absent Present	77, (78.6) 20, (20.4)	0.21 (0.03–1.75)	0.150
**Debulking status** Optimal Suboptimal	71, (72.4) 27, (27.6)	1.38 (0.34–5.55)	0.651
**Response to Therapy** Complete Response Other Responses	54, (55.1) 20, (20.4)	0.71 (0.23–2.26)	0.568
**Platinum sensitivity** Yes No	48, (49.0) 27, (27.6)	1.07 (0.34–3.31)	0.907

N = 98 patients with high grade serous ovarian cancer. Multivariate logistic regression analysis showed a significant association (odds ratio 4.70; 95% confidence interval, 1.48–14.97) between age ≥ 70 years and presence of VOI in a specimen.

### Association between virus of interest and overall survival

**Fig 1:**
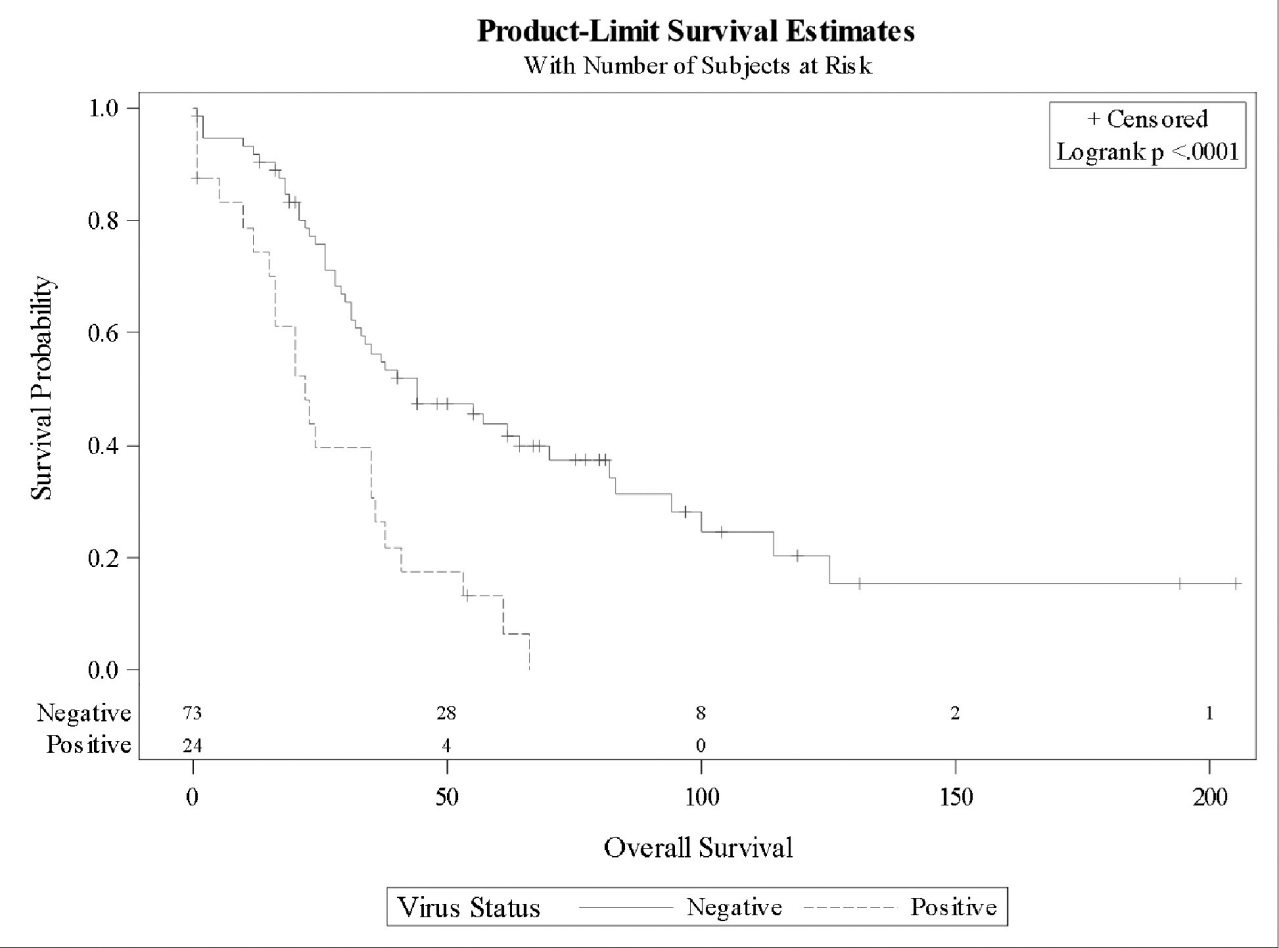
Kaplan Meier curves portraying overall survival for cases with tumor specimen having a virus of interest (VOI) versus no VOI. The median overall survival for cases with VOI was 22 months (15–35 months) versus 44 months for cases without VOI (31–70 months). Log-rank *P* < 0.0001.

**Fig 2:**
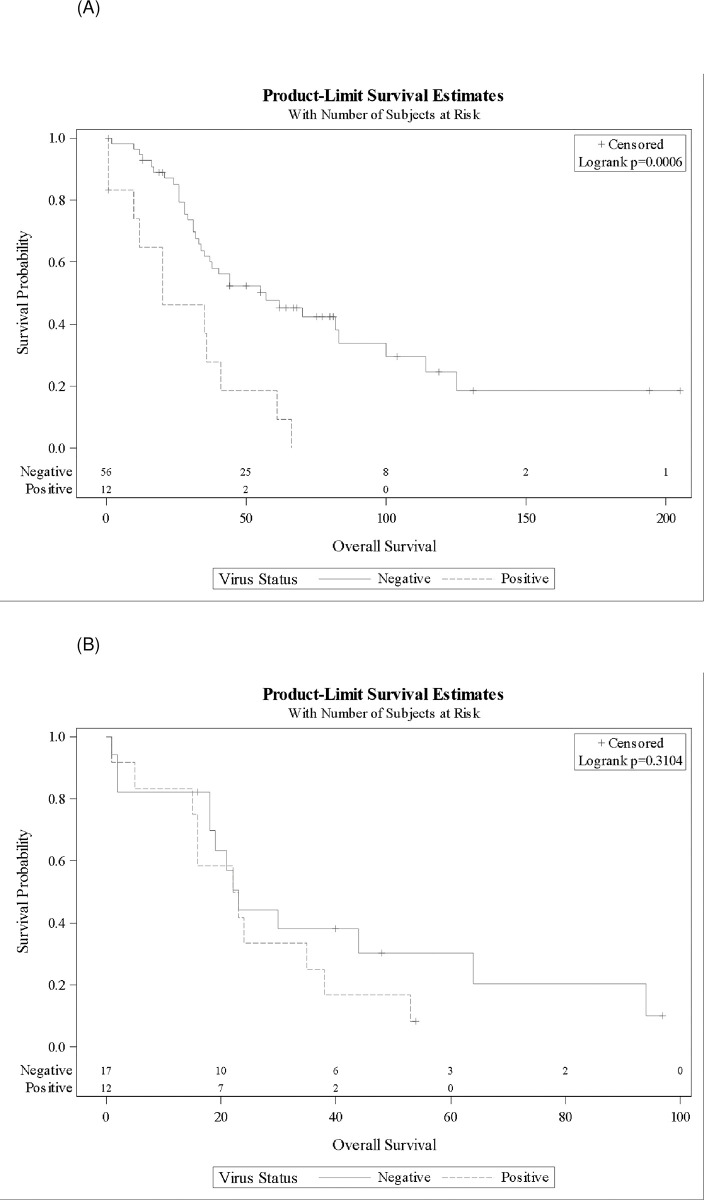
(A) Kaplan-Meier curves portraying overall survival for patients less than 70 years old whose tumor specimen had a virus of interest (VOI) versus no VOI. The median overall survival for patients with VOI was 20 months (1–41 months) versus 57 months for those without VOI (34–83 months). Log-rank *P* = 0.0006. (B) Kaplan-Meier curves portraying overall survival for patients 70 years and older whose tumor specimen had a VOI versus no VOI. The median overall survival for patients with VOI was 22.5 months (5–38 months) versus 23 months for those without VOI (18–64 months). Log-rank *P* = 0.31.

Survival analyses indicated no differences in outcomes for patients with a specimen containing no viral DNA or in those without a VOI (low prevalence viruses). These groups were combined for subsequent analyses (Kaplan-Meier survival analyses). In multivariate Cox proportional hazard models, VOI was associated with a four-fold lower OS (HR 4.11, *P* = 0.0001). Older age and advanced stage at diagnosis were also significantly associated with poorer OS (Table 2). Expectedly, platinum-sensitive disease was associated with a survival advantage (HR 0.21, *P*<0.0001). The median OS was significantly reduced in the presence of VOI versus not having a VOI (22 vs 44 months, *P*<0.0001) (Fig 1). In a sub-analysis of women < 70 years old, the median OS was significantly decreased for patients with a VOI compared with those not having a VOI (20 vs 57 months, *P* = 0.0006); however, among women ≥ 70 years old, there was no difference in OS by tumor viral status (Fig 2A and 2B respectively).

**Table 2:** Factors independently associated with poorer overall survival in a multivariate cox proportional hazard model.

Variable	Hazard Ration (95% CI)	P Value
Virus of Interest*	4.11 (2.00–8.44)	0.0001
Age**	1.85 (0.97–3.53)	0.06
Stage^	5.77 (0.78–42.73)	0.09
Platinum Sensitivity^#^	0.21 (0.11–0.40)	<0.0001

*Virus of interest vs no virus of interest.

**Age ≥70 years vs < 70 years.

^Advanced (III/IV) vs early (I/II) stage.

^#^Platinum sensitive vs platinum resistant or refractory disease are independently associated with poorer overall survival.

## Discussion

In this study, we examined the prevalence of 113 specific viruses from three viral families (herpesviridae, polyomaviridae, and papillomaviridae). In our cohort of 98 SEOC specimens, we found that the overall prevalence of viral DNA was 46.9%. Our study focused on SEOC because it is the most common histology among ovarian cancer. To our knowledge, this is the most comprehensive panel of viral DNA evaluated in ovarian cancer tumor specimens and the highest reported prevalence of viral DNA in SEOC specimens from a North American cohort. Furthermore, DNA from known or suspected oncogenic viruses was found in a significant proportion of the SEOC samples (24.5%). Importantly, we found that the presence of 6 viruses, which we termed VOI, substantially reduced the median OS time.

The number and type of oncogenic viruses found in these tumor samples raise important questions about the implications of the presence of viral DNA in ovarian cancer specimens. Is the viral DNA found in ovarian cancer tumor samples merely an inactive passenger or contaminant, or do these VOIs modulate tumor biology or alter the host-tumor microenvironment in such a manner as to affect clinical outcomes? The available literature regarding viruses in malignant tumors (ovarian and other cancers) suggests that viruses preferentially bind to and infect tumor versus normal tissue [8] and that tumors harboring viruses have alterations in the immune microenvironment [17–24].

A publication by Kines et al. examined the ability of HPV capsids to bind and infect malignant ovarian tissues in a mouse model [8]. This group reported a preference of the viral capsids for malignant tumor tissue compared with adjacent normal tissue. This affinity for malignant tissue was attributed to alterations in tumor heparin sulfate proteoglycans, the cell-entry binding site for the viral capsids, suggesting that viruses preferentially bind malignant ovarian tumors.

Pandya et al., who examined the prevalence of viral microRNAs in a Cancer Genome Atlas cohort of malignant ovarian tumors, reported a higher prevalence of viral microRNAs, specifically microRNAs from HHV6VB and HSV2, in malignant ovarian tissue than in normal tissue [9]. Furthermore, the authors reported that the presence of microRNA-BART7 from EBV is associated with platinum resistance and worsened survival. These data are in agreement with our finding of decreased median OS for tumors containing certain viral DNA.

Ovarian tumors have an intimate interaction with host immune cells. The prognostic values of tumor-infiltrating immune cell lineages [17–24], major histocompatibility complex (MHC) expression [25–27], and immune checkpoint protein expression [28–30] are well documented. Although not previously evaluated in ovarian cancer, viruses can modulate immune function. Hatam et al. reported that HPV-induced premalignant respiratory papillomas express the regulatory T cell (Treg) chemoattractant CCL17 and express PD-L1, whereas autologous control laryngeal tissues did not [31]. A comparison of tumor samples from patients with hepatocellular carcinoma of hepatitis B origin (HBVHCC) and non-HBVHCC suggested that HBVHCC tissues had higher concentrations of Tregs and decreased numbers of CD8-positive T cells compared with non-HBVHCC tissues [32]. Additionally, in a study evaluating oropharyngeal squamous cell carcinoma, HPV-positive tumors were more likely to express PD-L1, which correlated with distant metastases [33]. Furthermore, in a study of premalignant cervical dysplasia, Molling and associates reported higher Treg frequencies in patients with persistent HPV infection and noted that Treg numbers were increased in samples with detectable HPV16 E7-specific T-helper cells compared with samples where these cells were not detected [34]. Several virus species can modulate the expression and function of MHC. A recent study of MCPyV indicated that, compared with adjacent normal tissues and polyomavirus-negative samples, MCPyV-positive samples had reduced expression of MHC class I [35]. Furthermore, although the mechanisms seem to vary, other viruses, including HPV [36–38], EBV1 [39, 40], and HHV6B [41, 42], directly or indirectly downregulate MHC class I expression and/or interfere with antigen presentation. We hypothesize that the immune environment differs in tumor samples and differs between those with and without VOI and that the differences in the immune microenvironment may contribute to the observed survival differences. Due to the limited availability of additional tumor tissue, further determination of the status of CD8 and PD-L1 was not possible in this study. Therefore, the potential impact of these viruses on immunomodulation in the tumor environment remains unexplored.

The strengths of this study include the utilization of a widely validated platform for the detection of viral DNA. Furthermore, to our knowledge, this is the most comprehensive viral panel examined in ovarian tumor specimens. The use of banked tissues raises the question of possible contamination during storage. However, all tumor specimens and clinical data were collected, processed, and stored by Moffitt’s TCC® institutional clinic-genomic tissue and data repository, which is highly regulated and robust.

In conclusion, 46.5% of our SEOC specimens contained viral DNA, with presence of VOIs associated with a significantly worsened median OS. The literature clearly supports the importance of the host-tumor immune microenvironment’s relationship to clinical outcomes as well as the potential for viruses to modulate this intricate system. Further work is needed to understand how the presence of viruses in ovarian cancer tumors influences host-tumor immune interactions and ultimately impacts clinical outcomes.

## Supporting information

S1 Checklist STROBE statement—checklist of items that should be included in reports of observational studies. (DOCX)
