# Supplementary material for: Prevalence of viral DNA in high-grade serous epithelial ovarian cancer and correlation with clinical outcomes
Source: PLoS One. 2023 Dec 1;18(12):e0294448. doi: 10.1371/journal.pone.0294448 (PMC10691703; doi:10.1371/journal.pone.0294448)
Supplement: S1 Checklist — (DOCX) [file pone.0294448.s001.docx]

STROBE Statement—checklist of items that should be included in reports of observational studies

|  | Item No. | Recommendation | Page  No. | Relevant text from manuscript |
| --- | --- | --- | --- | --- |
| **Title and abstract** | 1 | (*a*) Indicate the study’s design with a commonly used term in the title or the abstract | 2 | This is a retrospective cohort study using archived tumor samples from 98 patients diagnosed with high-grade serous epithelial ovarian cancer were collected between 1/1/1994 and 12/31/2010. |
|  |  | (*b*) Provide in the abstract an informative and balanced summary of what was done and what was found | 2. | Forty-six cases (45.9%) contained at least one virus. Six highly prevalent viruses were associated with clinical outcomes and considered viruses of interest (VOI; Epstein-Barr virus 1, Merkel cell polyomavirus, human herpes virus 6b, and human papillomaviruses 4, 16, and 23). Factors independently associated with OS were presence of VOI (HR 4.11, *P*=0.0001) and platinum sensitivity (HR 0.21, *P*<0.0001). Median OS was significantly decreased when tumors showed VOI versus not having these viruses (22 vs 44 months, *P*<0.0001). |
| Introduction | | | |  |
| Background/rationale | 2 | Explain the scientific background and rationale for the investigation being reported | 4 | Eleven infectious agents are classified as carcinogenic by the International Agency for Research on Cancer.  Ovarian cancer, the most lethal gynecologic malignancy does not have a known infectious etiology.  Pandya et al reported that the expression of viral microRNAs in ovarian cancer tissues is higher than expression in control tissues and that expression of specific microRNAs correlates with clinical outcome [9]. These data suggest that malignant ovarian tumors contain viruses, and these viruses have the potential to affect clinical outcome. |
| Objectives | 3 | State specific objectives, including any prespecified hypotheses | 4 | Here, we investigated the prevalence of viral DNA in epithelial ovarian cancer in a cohort from a single institution and assessed the association between presence of viral DNA and clinical outcomes. |
| Methods | | | |  |
| Study design | 4 | Present key elements of study design early in the paper | 5 | This is a retrospective cohort study. |
| Setting | 5 | Describe the setting, locations, and relevant dates, including periods of recruitment, exposure, follow-up, and data collection | 5 | The patient samples and molecular and clinical data from high-grade serous epithelial ovarian cancer (SEOC) diagnosed between January 1, 1994, and December 31, 2020l were retrieved from the the Moffitt Cancer Center Total Cancer Care (TCC®) institutional clinico-genomic tissue and data repository after obtaining IRB approval from the University of South Florida. All patients included in the TCC protocol (Liberty IRB no. 00014441) provided written informed consent prospectively. Tumor samples were originally collected at the time of primary cytoreductive surgery. Tumor samples that had been exposed to a chemotherapeutic agent before surgery, a non-serous, low-grade, or borderline histology were also excluded. After samples were retrieved from the TCC repository, patient identifiers were used for chart abstraction to collect demographic data (age at cancer diagnosis and race/ethnicity), disease characteristics (cancer stage, presence of ascites, pleural effusion, and lymph node metastasis), treatment characteristics (status of surgical cytoreduction, response to first-line chemotherapy, and platinum-sensitive disease), and survival data. To endure privacy and confidentiality, patient identifiers were securely separated from the dataset prior to data analysis. |
| Participants | 6 | (*a*) *Cohort study*—Give the eligibility criteria, and the sources and methods of selection of participants. Describe methods of follow-up  *Case-control study*—Give the eligibility criteria, and the sources and methods of case ascertainment and control selection. Give the rationale for the choice of cases and controls  *Cross-sectional study*—Give the eligibility criteria, and the sources and methods of selection of participants | 5 | The patient samples and molecular and clinical data from high-grade serous epithelial ovarian cancer (SEOC) diagnosed between January 1, 1994, and December 31, 2020l were retrieved from the the Moffitt Cancer Center Total Cancer Care (TCC®) institutional clinico-genomic tissue and data repository after obtaining IRB approval from the University of South Florida. All patients included in the TCC protocol (Liberty IRB no. 00014441) provided written informed consent prospectively. Tumor samples were originally collected at the time of primary cytoreductive surgery. Tumor samples that had been exposed to a chemotherapeutic agent before surgery, a non-serous, low-grade, or borderline histology, incomplete data were also excluded. |
|  |  | (*b*) *Cohort study*—For matched studies, give matching criteria and number of exposed and unexposed  *Case-control study*—For matched studies, give matching criteria and the number of controls per case |  | NA |
| Variables | 7 | Clearly define all outcomes, exposures, predictors, potential confounders, and effect modifiers. Give diagnostic criteria, if applicable | 7  8  9 | Prevalance of virus  Clinical characteristics  Association between virus of interest and overall survival |
| Data sources/ measurement | 8* | For each variable of interest, give sources of data and details of methods of assessment (measurement). Describe comparability of assessment methods if there is more than one group | 6  5,6  7 | **Prevalance of virus** -  The identification of viral DNA was performed at the International Agency for Research on Cancer. Formalin-fixed paraffin-embedded SEOC tissue blocks were obtained from Moffitt’s tissue repository. Genomic DNA (400 ng) was extracted from these specimens using standard techniques. DNA was amplified by a multiplex polymerase chain reaction (PCR) protocol and identified as belonging to one of 113 infectious agents, including 93 HPVs, 10 polyomaviruses, and 8 herpesviruses, as well as the bacterium *Chlamydia trachomatis* using Luminex technology  **Clinical characteristics**  After samples were retrieved from the TCC repository, patient identifiers were used for chart abstraction to collect demographic data (age at cancer diagnosis and race/ethnicity), disease characteristics (cancer stage, presence of ascites, pleural effusion, and lymph node metastasis), treatment characteristics (status of surgical cytoreduction, response to first-line chemotherapy, and platinum-sensitive disease), and survival data. To endure privacy and confidentiality, patient identifiers were securely separated from the dataset prior to data analysis.  **Association between virus of interest and overall survival**  Cox proportional hazard model was used to assess the independent association between viral DNA presence and overall survival (OS). |
| Bias | 9 | Describe any efforts to address potential sources of bias | 12 | The use of banked tissues raises the question of possible contamination during storage. However, all tumor specimens and clinical data were collected, processed, and stored by Moffitt’s TCC® institutional clinic-genomic tissue and data repository, which is highly regulated and robust. |
| Study size | 10 | Explain how the study size was arrived at | 7 | We initially identified 101 cases of high-grade epithelial ovarian cancer. After exclusion of 3 samples that were non-serous histology, 98 samples were available for analysis, with 46 of these specimens (46.9%) containing DNA from at least one virus.. |

Continued on next page

| Quantitative variables | 11 | Explain how quantitative variables were handled in the analyses. If applicable, describe which groupings were chosen and why | 7  8 | 98 samples were available for analysis, with 46 of these specimens (46.9%) containing DNA from at least one virus. Multiple viral infections were found in one tumor specimen that tested positive for two beta HPV types (HPV23 and HPV111), one gamma HPV type (HPV123), and one herpesvirus (HHV6B). Two herpesviruses (EBV1 and HHV6b), one polyomavirus (MCPyV), one gamma HPV type (HPV4), one beta HPV type (HPV23), and one mucosal high-risk HPV type 16 (HPV16) were the six most prevalent viruses.  DNA from 5 of the prevalent viruses (EBV1, HHV6B, MCPyV, HPV4, and HPV16) were each identified in four unique tumor specimens (4.1%), whereas HPV23 viral DNA was identified in nine tumor specimens (9.2%). Preliminary survival analyses suggested that patients with tumor samples containing one or more of the highly prevalent viruses had significantly worse OS than patients with tumors containing viral DNA that was not highly prevalent or tumors without any viral DNA. These highly prevalent viruses (EBV1, MCPyV, HHV6b, HPV4, HPV16, and HPV23) were therefore grouped and considered viruses of interest (VOI) for the purposes of subsequent analyses. |
| --- | --- | --- | --- | --- |
| Statistical methods | 12 | (*a*) Describe all statistical methods, including those used to control for confounding | 7-9 | Prevalence of virus   - Descriptive statistics were performed   Clinical characteristic  Descriptive statistics were performed for demographic data and disease characteristics. Logistic regression was performed to assess the association between the presence of viral DNA and clinical characteristics.  Association between virus of interest and overall survival  Cox proportional hazard model was used to assess the independent association between viral DNA presence and overall survival (OS). A backward model selection was used for both logistic regression and the Cox proportional hazard models. All variables significant at *P* ≤ 0.1 remained in the final model. |
|  |  | (*b*) Describe any methods used to examine subgroups and interactions |  | NA |
|  |  | (*c*) Explain how missing data were addressed |  | NA |
|  |  | (*d*) *Cohort study*—If applicable, explain how loss to follow-up was addressed  *Case-control study*—If applicable, explain how matching of cases and controls was addressed  *Cross-sectional study*—If applicable, describe analytical methods taking account of sampling strategy |  | NA |
|  |  | (*e*) Describe any sensitivity analyses |  | NA |
| Results | | | | |
| Participants | 13* | (a) Report numbers of individuals at each stage of study—eg numbers potentially eligible, examined for eligibility, confirmed eligible, included in the study, completing follow-up, and analysed | 7 | We initially identified 101 cases of high-grade epithelial ovarian cancer. After exclusion of 3 samples that were non-serous histology, 98 samples were available for analysis. |
|  |  | (b) Give reasons for non-participation at each stage |  | NA |
|  |  | (c) Consider use of a flow diagram |  | NA |
| Descriptive data | 14* | (a) Give characteristics of study participants (eg demographic, clinical, social) and information on exposures and potential confounders |  | See table 1 |
|  |  | (b) Indicate number of participants with missing data for each variable of interest |  | NA |
|  |  | (c) *Cohort study*—Summarise follow-up time (eg, average and total amount) |  | NA |
| Outcome data | 15* | *Cohort study*—Report numbers of outcome events or summary measures over time |  | *NA* |
|  |  | *Case-control study—*Report numbers in each exposure category, or summary measures of exposure |  | *NA* |
|  |  | *Cross-sectional study—*Report numbers of outcome events or summary measures |  | *NA* |
| Main results | 16 | (*a*) Give unadjusted estimates and, if applicable, confounder-adjusted estimates and their precision (eg, 95% confidence interval). Make clear which confounders were adjusted for and why they were included |  | NA |
|  |  | (*b*) Report category boundaries when continuous variables were categorized |  | NA |
|  |  | (*c*) If relevant, consider translating estimates of relative risk into absolute risk for a meaningful time period |  | NA |

Continued on next page

| Other analyses | 17 | Report other analyses done—eg analyses of subgroups and interactions, and sensitivity analyses |  | NA |
| --- | --- | --- | --- | --- |
| Discussion | | | | |
| Key results | 18 | Summarise key results with reference to study objectives | 9, 10 | In this study, we examined the prevalence of 113 specific viruses from three viral families (herpesviridae, polyomaviridae, and papillomaviridae). In our cohort of 98 SEOC specimens, we found that the overall prevalence of viral DNA was 46.9%. To our knowledge, this is the most comprehensive panel of viral DNA evaluated in ovarian cancer tumor specimens and the highest reported prevalence of viral DNA in SEOC specimens from a North American cohort. Furthermore, DNA from known or suspected oncogenic viruses was found in a significant proportion of the SEOC samples (24.5%). Importantly, we found that the presence of 6 viruses, whic |
| Limitations | 19 | Discuss limitations of the study, taking into account sources of potential bias or imprecision. Discuss both direction and magnitude of any potential bias | 12 | The strengths of this study include the utilization of a widely validated platform for the detection of viral DNA. Furthermore, to our knowledge, this is the most comprehensive viral panel examined in ovarian tumor specimens. The use of banked tissues raises the question of possible contamination during storage. However, all tumor specimens and clinical data were collected, processed, and stored by Moffitt’s TCC® institutional clinic-genomic tissue and data repository, which is highly regulated and robust. |
| Interpretation | 20 | Give a cautious overall interpretation of results considering objectives, limitations, multiplicity of analyses, results from similar studies, and other relevant evidence | 11,12 | The number and type of oncogenic viruses found in these tumor samples raise important questions as to implications of the presence of viral DNA in ovarian cancer specimens. Is the viral DNA found in ovarian cancer tumor samples merely an inactive passenger or contaminant, or do these VOIs modulate tumor biology or alter the host-tumor microenvironment in such a manner as to affect clinical outcomes? The available literature regarding viruses in malignant tumors (ovarian and other cancers) suggests that viruses preferentially bind to and infect tumor versus normal tissue [8] and that tumors harboring viruses have alterations in the immune microenvironment [17-24].  A publication by Kines et al examined the ability of HPV capsids to bind and infect malignant ovarian tissues in a mouse model [8]. This group reported a preference of the viral capsids for malignant tumor tissue compared with adjacent normal tissue. This affinity for malignant tissue was attributed to alterations in tumor heparin sulfate proteoglycans, the cell-entry binding site for the viral capsids, suggesting that viruses preferentially bind malignant ovarian tumors.  Pandya et al, who examined the prevalence of viral microRNAs in a Cancer Genome Atlas cohort of malignant ovarian tumors, reported a higher prevalence of viral microRNAs, specifically microRNAs from HHV6VB and HSV2, in malignant ovarian tissue than in normal tissue [9]. Furthermore, the authors reported that the presence of microRNA-BART7 from EBV is associated with platinum resistance and worsened survival. These data are in agreement with our finding of decreased median OS for tumors containing certain viral DNA.  Ovarian tumors have an intimate interaction with host immune cells. The prognostic values of tumor-infiltrating immune cell lineages [17-24], major histocompatibility complex (MHC) expression [25-27], and immune checkpoint protein expression [28-30] are well documented. Although not previously evaluated in ovarian cancer, viruses can modulate immune function. Hatam et al. reported that HPV-induced premalignant respiratory papillomas express the regulatory T cell (Treg) chemoattractant CCL17 and express PD-L1, whereas autologous control laryngeal tissues did not [31]. A comparison of tumor samples from patients with hepatocellular carcinoma of hepatitis B origin (HBVHCC) and non-HBVHCC suggested that HBVHCC tissues had higher concentrations of Tregs and decreased numbers of CD8-positive T cells compared with non-HBVHCC tissues [32]. Additionally, in a study evaluating oropharyngeal squamous cell carcinoma, HPV-positive tumors were more likely to express PD-L1, which correlated with distant metastases [33]. Furthermore, in a study of premalignant cervical dysplasia, Molling and associates reported higher Treg frequencies in patients with persistent HPV infection and noted that Treg numbers were increased in samples with detectable HPV16 E7-specific T-helper cells compared with samples where these cells were not detected [34]. Several virus species can modulate the expression and function of MHC. A recent study of MCPyV indicated that, compared with adjacent normal tissues and polyomavirus-negative samples, MCPyV-positive samples had reduced expression of MHC class I [35]. Furthermore, although the mechanisms seem to vary, other viruses, including HPV [36-38], EBV1 [39, 40], and HHV6B [41, 42], directly or indirectly downregulate MHC class I expression and/or interfere with antigen presentation. We hypothesize that the immune environment differs in tumor samples and differs between those with and without VOI and that the differences in the immune microenvironment may contribute to the observed survival differences. |
| Generalisability | 21 | Discuss the generalisability (external validity) of the study results | 12 | The literature clearly supports the importance of the host-tumor immune microenvironment’s relationship to clinical outcomes as well as the potential for viruses to modulate this intricate system. Further work is needed to understand how the presence of viruses in ovarian cancer tumors influences host-tumor immune interactions and ultimately impacts clinical outcomes. |
| Other information | |  | | |
| Funding | 22 | Give the source of funding and the role of the funders for the present study and, if applicable, for the original study on which the present article is based | 13 | This work has been supported in part by Moffitt’s Total Cancer Care Initiative and the Collaborative Data Services Core at the H. Lee Moffitt Cancer Center & Research Institute, an NCI designated Comprehensive Cancer Center, under grant number P30-CA076292. A. R. Giuliano received funding from National Institutes of Health Grant K05-CA181320-04. |

*Give information separately for cases and controls in case-control studies and, if applicable, for exposed and unexposed groups in cohort and cross-sectional studies.

**Note:** An Explanation and Elaboration article discusses each checklist item and gives methodological background and published examples of transparent reporting. The STROBE checklist is best used in conjunction with this article (freely available on the Web sites of PLoS Medicine at http://www.plosmedicine.org/, Annals of Internal Medicine at http://www.annals.org/, and Epidemiology at http://www.epidem.com/). Information on the STROBE Initiative is available at www.strobe-statement.org.
